# The sense of agency in near and far space: where do we stand?

**DOI:** 10.1093/nc/niaf066

**Published:** 2026-01-27

**Authors:** Gaiqing Kong, Marine Vernet, Alessandro Farnè

**Affiliations:** Department of Psychology, School of Sports Medicine, Wuhan Sports University, No. 461 Luoyu Road, Hongshan District, Wuhan, Hubei Province 430079, China; Impact Team, Lyon Neuroscience Research Centre (CRNL), INSERM 1028 – CNRS UMR5292, University Claude Bernard Lyon 1, 16 Av. du Doyen Jean Lépine, 69500 Bron, France; Impact Team, Lyon Neuroscience Research Centre (CRNL), INSERM 1028 – CNRS UMR5292, University Claude Bernard Lyon 1, 16 Av. du Doyen Jean Lépine, 69500 Bron, France; Impact Team, Lyon Neuroscience Research Centre (CRNL), INSERM 1028 – CNRS UMR5292, University Claude Bernard Lyon 1, 16 Av. du Doyen Jean Lépine, 69500 Bron, France; Neuro-Immersion, Lyon Neuroscience Research Centre (CRNL), INSERM 1028 – CNRS UMR5292, University Claude Bernard Lyon 1, 16 Av. du Doyen Jean Lépine, 69500 Bron, France

**Keywords:** sense of agency, spatial distance, spatial proximity, temporal binding, temporal interval estimation, explicit agency, action awareness

## Abstract

Modern technology frequently places the consequences of our actions at a distance (e.g. remote surgery, smart-home control, virtual reality). Does spatial distance between an action and its outcome weaken the sense of agency (SoA) - the feeling of control over one’s actions and consequences? Two recent studies, by Jenkins and Obhi and Mariano et al., answered “yes,” reporting stronger temporal binding (TB) in near than far space and interpreting this as greater implicit agency. A third study - our own work with a similar paradigm - found no distance effect. Here we (i) provide a rigorous side-by-side methodological comparison of the three studies, (ii) argue why a direct test to establish a distance modulation of TB (the Near - Far difference of the Active - Passive delta) should be performed in order to reach meaningful conclusions, and (iii) report new reanalyses of our data and direct tests on the two target studies. Overall, current evidence does not support a distance effect on SoA. Our reassessment provides alternative explanations that converge with available evidence suggesting that distance may influence temporal interval perception, but that effect is independent of action intention and therefore of agency.

**Public Significance Statement**: In our increasingly connected world, we often interact with devices and influence events that are physically distant from us - like controlling smart appliances remotely or engaging in virtual reality experiences. Does the distance between us and the effects of our actions change how much we feel in control of the outcomes of our actions? Recent studies have suggested that we feel less responsible when action outcomes appear in far space compared to near space. However, methodological limitations in these studies, as well as our own results with a similar paradigm, challenge the validity of this claim. We aim to contribute to the crucial debate on the role of spatial distance on humans’ feeling of responsibility by providing a respectful though critical analysis of recent findings and offering recommendations for future research.

## Introduction

Modern technology has dramatically transformed how we interact with the world, enabling actions performed in near space to produce far-reaching consequences. Despite this shift, the role of spatial distance - specifically where the outcomes of our actions are perceived - has been largely overlooked in research on the sense of agency (SoA). SoA is a fundamental component of self-consciousness, allowing individuals to recognize themselves as the agents of their actions and their ensuing consequences (Haggard and Chambon 2012, Haggard 2017). Understanding how spatial context influences SoA is particularly relevant in today’s increasingly digital and remote environments, where the boundaries between near and far space are becoming increasingly blurred. However, this question has remained largely underexplored in previous research.

Mariano et al. (2024) sought to address this question by manipulating the spatial context in which participants perceived their action outcomes, using both illusory computer-based and realistic settings. In their study, participants performed an active or passive button press to light up a light bulb. The authors used an implicit measure of agency, the “intentional binding” (IB), which refers to the phenomenon where individuals perceive the temporal interval between their voluntary action and its consequence as shorter than the interval between two events in which they are not voluntarily involved. The authors reported that such binding was greater when outcomes occurred in near space compared to far space, regardless of the ecological validity of the setting. In contrast, explicit agency judgments were not significantly affected by spatial distance. The authors concluded that spatial proximity - whether realistic or illusory - is essential for feeling implicitly responsible for the consequences of our actions.

Previously, Jenkins and Obhi (2021) also explored the relationship between perceived action–outcome distance and the sense of agency using the temporal binding effect, a more general term encompassing IB. In their online study, they manipulated the perceived near and far distances of action outcomes through perspective cues. Participants triggered the appearance of a circle at different perceived distances by a voluntary keypress or observed the same circle being triggered by a computer-generated tone. Jenkins and Obhi found that participants’ interval estimates - the perceived time between action and outcome - increased with the perceived distance of the outcome. However, this increase was not restricted to conditions involving active movement within a 3D perspective; it was also observed in other conditions. The authors carefully stated that their results point to a link between intentionality and distance, in the direction of a reduced sense of agency for outcomes that appear further away, while acknowledging the limitations of their study and the need for further research.

Having been researching on this topic without finding evidence for an effect of spatial proximity on “intentional binding” (Kong et al. 2024), we closely inspected and provided a direct reanalysis of the available work, including our own. Specifically, we analytically assess the work of both Mariano et al. (2024) and Jenkins and Obhi (2021), focusing on data analyses and the conclusions drawn, and we also compare their results with our own previous data (Kong et al. 2024), which did not support the effect of distance on temporal binding in contexts involving intentional movements. We end by suggesting directions for future research in this domain.

### How to measure temporal binding and assess its modulation by spatial proximity

Both Jenkins and Obhi (2021) and Mariano et al. (2024) investigated the effect of spatial distance on the SoA by manipulating the perceived location of action outcomes. Jenkins and Obhi manipulated spatial distance using visual perspective cues in an online study, presenting outcomes that appeared near or far from the participant’s viewpoint (Table 1). The spatial proximity of the outcome was cued using a 3D perspective (simulating near vs far space by varying the relative size and position of the visual elements). As a control, a flat 2D condition without perspective cues was used. Mariano et al. used both computer-based environments and realistic settings to present action outcomes in near (~35 cm) and far (~70 cm) space (Table 1). Both studies employed the temporal binding paradigm with the interval estimation procedure. In this paradigm, the perceived time interval between an action and its outcome is typically shortened for voluntary (active) movements compared to involuntary (passive) movements, or baseline conditions, and this temporal compression is taken as an implicit measure of SoA (Moore and Obhi 2012). Jenkins and Obhi (2021) employed intervals of 200, 500, and 800 ms, while Mariano et al. (2024) used intervals of 200, 400, and 600 ms.

**Table 1:** Three studies have brought temporal binding (TB) to bear on spatial distance

Study	Display and distance cue	First-event type	Delays (ms)	Core claim
**Jenkins** **and** **Obhi** **(2021)**	PC monitor; linear-perspective cues; *flat* “no-perspective” control	Active keypress; external event	200/500/800	200 ms intervals judged shorter when the circle appears “near”
**Mariano** **et al.** **(2024)**	(a) PC, retinal-size cue and (b) real table top, 35 vs 70 cm	Active keypress, passive keypress	200/400/600	Larger TB at 200 ms in near versus far space
**Kong** **et al.** **(2024)**	Immersive VR, binocular disparity (45 cm versus 4.5 m)	Active keypress, passive keypress, external event	150/450/750	Distance does not modulate TB

As a premise that we have addressed in previous work, we wish to note that, despite still being widely used, the intentional binding phenomenon is increasingly recognized as multifaceted and potentially confounded. Recent studies have shown that intentional binding may arise from factors such as causality, predictability, and somatosensory integration, rather than uniquely reflecting intentionality and the voluntariness of action per se (Buehner and Humphreys 2009, Buehner 2015, Kirsch et al. 2019, Suzuki et al. 2019, Gutzeit et al. 2023, Dewey 2024, Kong et al. 2024). Consequently, researchers should be cautious when using “intentional binding” as a reliable proxy for the sense of agency. Both Mariano et al. (2024) and Jenkins and Obhi (2021) acknowledge these criticisms. However, Mariano et al. (2024) argue that there are no alternative explanations, and state “the measure is achieved via the comparison of active intentional versus passive movements and it is systematically larger for active movements (i.e. shorter perceived temporal interval between actions and consequences)” (p. 2). Jenkins and Obhi (2021) note alternative interpretations that question whether intentional binding necessarily reflects agency, such as causal inference and multisensory integration. Yet, they also suggest that these criticisms are not definitive and that there remains evidence supporting temporal binding as a measure influenced by intentional actions. We concur that comparing a voluntary movement condition with an involuntary baseline condition is critical for measuring temporal binding (Haggard et al. 2002, Moore and Obhi 2012), though it may not alone justify labeling the effect as “intentional.” The importance of relying on the comparison between an active condition and a baseline forms the basis of our assessment of the two previous studies (Jenkins and Obhi 2021, Mariano et al. 2024).

Mariano et al. concluded that the intentional binding effect is larger in near space compared to far space, based on a pairwise comparison of active trials with a 200 ms action–outcome delay between near and far space (*t*(54) = −3.57, Bonferroni-corrected *P* = .05, Cohen’s *d* = −0.95). While passive trials were included in their analysis of variance (ANOVA), the conclusion was drawn from only comparing active trials between distances and passive trials between distances separately, which we deem problematic. Intentional binding is defined as the perceived shortening of the time interval between an action and its outcome when the action is intentional, compared to unintentional actions, or mere action observation. Therefore, measuring the intentional binding effect requires calculating the difference in temporal interval estimates between active and passive trials. When investigating the influence of the spatial distance of an action outcome on the SoA, it is essential to access a difference-of-differences (i.e. a delta measure):


\begin{equation*}{\mathrm{IB}}_{\mathrm{Near}}={\mathrm{Active}}_{\mathrm{Near}}-{\mathrm{Passive}}_{\mathrm{Near}}\end{equation*}

\begin{equation*}{\mathrm{IB}}_{\mathrm{Far}}={\mathrm{Active}}_{\mathrm{Far}}-{\mathrm{Passive}}_{\mathrm{Far}}\end{equation*}

A spatial modulation of binding therefore requires a statistical comparison of IB_Near_ versus IB_Far_, rather than comparing active-only and/or passive-only estimates across distances.

**Figure 1:**
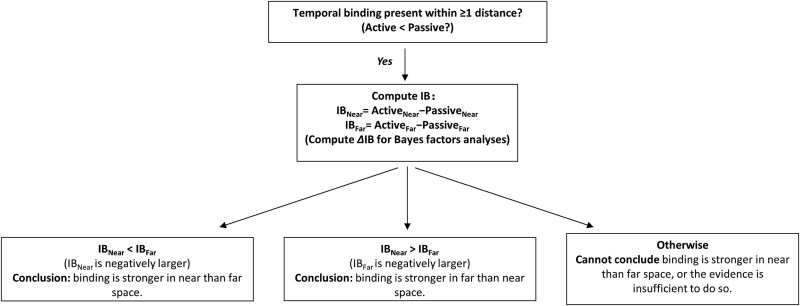
Decision tree for testing whether spatial distance modulates the temporal binding effect. First, establish a binding effect within at least one distance. Then, compute the binding effects for Near and Far, respectively, and statistically compare IB_Near_ and IB_Far_ using a null-hypothesis significance testing approach. For Bayesian inference, assess the modulation directly via the ΔIB (difference-of-differences) contrast. We would like to note: “significant in Near, non-significant in Far” is not evidence for a modulation; only a supported nonzero ΔIB warrants the conclusion (Gelman and Stern 2006, Pálfi and Dienes 2020).

Even when a significant Action × Distance interaction is observed in an ANOVA, this alone does not justify concluding a distance effect on binding. Such a conclusion requires specific *post hoc* tests confirming that the difference between active and passive trials is significantly larger in near space compared to far space (Fig. 1). Indeed, the values reported in Mariano et al. indicate that the actual difference in intentional binding (ΔIB) between near and far space is modest - ~11 ms in the computer-based condition and only ~1 ms in the realistic setting. Crucially, this difference was not directly tested: the well-established method for measuring IB, where each condition must be assessed relative to its corresponding baseline (Haggard et al. 2002), was not applied.

Taking this opportunity, we also wish to clarify the repeated use and the definition of the term “time compression” in some studies (Zapparoli et al. 2020a, 2020b, Mariano et al. 2024) to avoid misunderstanding. Defining “time compression” (TC) as the raw difference between the estimated and objective physical interval does not correspond to temporal binding (see Box 1). Such a quantity is more accurately described as a judgment error or a perceptual shift (Haggard et al. 2002, Kühn et al. 2013, Kong et al. 2017). Again, IB requires comparing active versus appropriate baseline conditions; absolute estimation error is not equivalent to IB. It is also worth noting another misinterpretation, particularly common in the intentional binding literature, namely, that IB measures the implicit sense of agency (see Box 1). This distinction is worth noting, as it appears increasingly frequently in the literature (Mariano et al. 2024, Zapparoli et al. 2020a, 2020b).

**Box 1: Two common misinterpretations in the IB literature**

**Misinterpretation 1: Difference between “temporal compression” and “temporal binding”.** Some studies define “temporal compression” (TC) as the raw difference between the actual duration and the estimated duration of the action–outcome delay and equate this quantity with IB. However, this quantity reflects “estimation/judgment error” or “perceptual shift/bias,” not IB. Intentional binding is the reduction in the perceived interval for voluntary actions relative to an appropriate baseline. Therefore, this absolute estimation error is not equal to IB. Humans systematically misjudge intervals (longer or shorter) in many contexts.

**Misinterpretation 2: Difference between “implicit measure of the sense of agency” and “measure of the implicit sense of agency”.**

It is worth noting a common misconception, particularly widespread in the IB literature, namely, that IB measures the implicit sense of agency. The measure is often described as “implicit” because it does not involve explicit reflection on agency; however, this does not necessarily mean that it measures an implicit agency.

Jenkins and Obhi (2021) also reported a distance effect, finding that the 200 ms interval was estimated to be shorter in near space compared to far space. However, this distance effect occurred irrespective of the presence or absence of intentional movement and was not directly linked to the temporal binding effect they observed at the shortest 200 ms delay. When all the delays were combined in the 3D condition, intervals were estimated to be shorter in near space than far space for the active movement task but not for the baseline task. However, no temporal binding was detected in the first place when comparing the active movement to the baseline tasks when all delays were combined in the 3D condition. Furthermore, the distance effect was also present in other conditions, such as the flat, no-perspective condition. The authors acknowledged these limitations and raised caution when suggesting that agency is reduced for outcomes that appear further away. Indeed, this conclusion requires further evidence.

In summary, we argue that any claim that spatial distance modulates intentional binding—and by extension, the sense of agency—requires the following minimal criteria (Fig. 1): (i) presence of a temporal binding effect: Active < Passive within at least one spatial condition; (ii) a specific comparison of binding magnitude across spatial conditions, i.e. (Active_Near_ − Passive_Near_) differs from (Active_Far_ − Passive_Far_) (Gelman and Stern 2006, Palfi and Dienes 2020). Previous work claiming a distance effect on SoA (Jenkins and Obhi 2021, Mariano et al. 2024) did not meet all of these criteria.

Finally, the issue is not only methodological but also theoretical: the passive condition serves as a baseline and its subtraction ensures that any observed effects would reflect changes in binding rather than general differences in time perception. We contend that the distance effect reported may reflect differences in temporal interval perception between the near and far conditions, rather than a spatial modulation of temporal binding. Below, reanalyses of previously published datasets directly assess the evidence for each of these competing hypotheses.

### Direct test: does spatial distance really modulate temporal binding?

**Figure 2:**
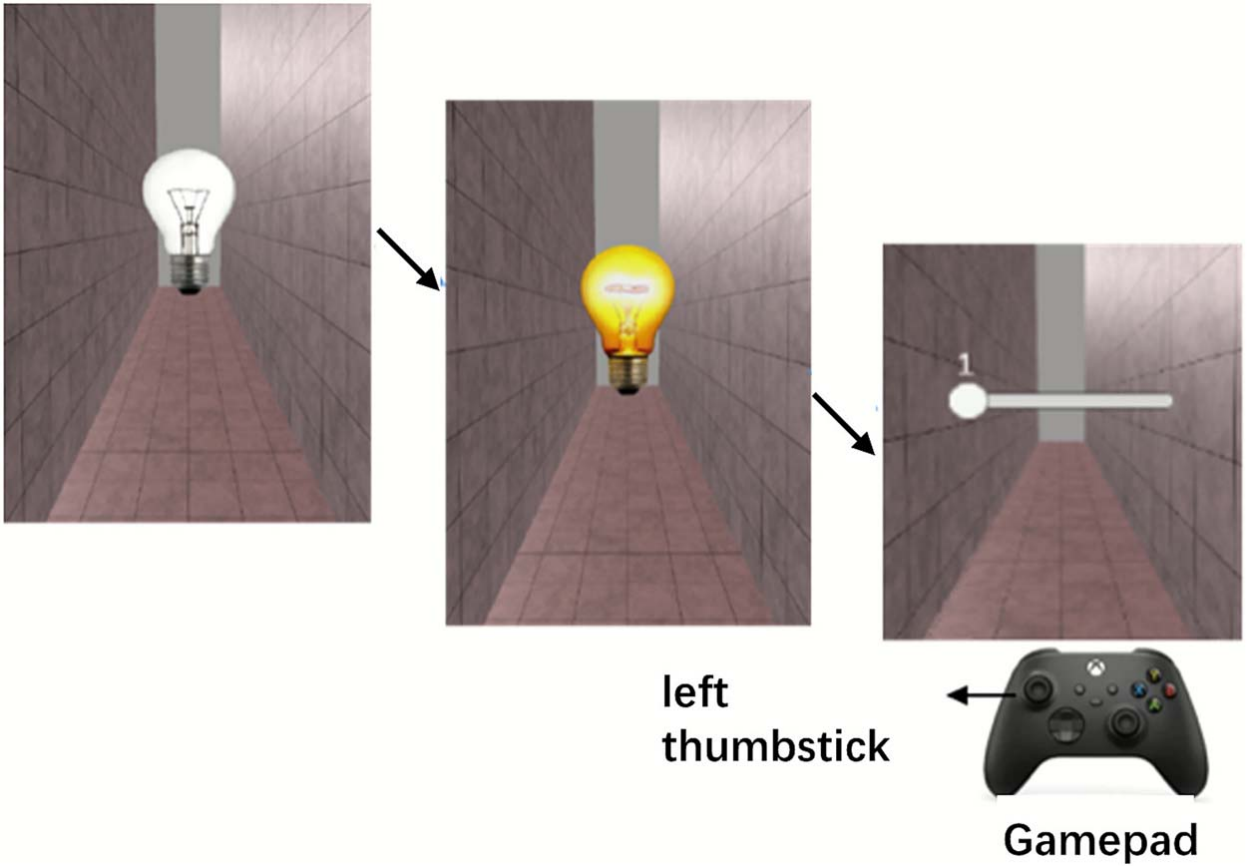
Experimental setup in our study. In our experiment (Kong et al. 2024), participants viewed a virtual scene through a head-mounted display (Oculus Rift CV1). The action outcome was a visual effect (the lighting of a light bulb) which was presented at two different distances: near (45 cm) and far (4.5 m). During each trial, an unlit bulb was turned on following different first events depending on the task: an active or passive keypress, or an external sensory event (the appearance of an off bulb). In the active movement task, participants performed a voluntary keypress with their left index finger whenever they wanted. In the passive movement task, the experimenter pressed the participant’s left index finger down onto the button. The keypress was performed using a gamepad (button “A” on the gamepad). After the lit bulb had appeared for one second, participants were asked to estimate the interval between the first event and the onset of the bulb lighting. The three possible intervals were randomly set to 150, 450, or 750 ms. Participants indicated their estimates by moving a visually depicted slider with the left thumbstick of the gamepad. The slider ranged from a minimum value of 1 ms to a maximum of 1000 ms, with the current value displayed in real time as participants adjusted it. The near and far bulbs were displayed with retinal correction.

Similar to previous work, in a recent study (Kong et al. 2024) we employed an immersive virtual reality (VR) environment to manipulate the location of a light bulb, presenting action outcomes (the bulb lightening) at 45 cm (near) and 4.5 m (far) (Table 1 and Fig. 2). The three delayed intervals tested were 150, 450, and 750 ms. In an attempt to isolate the role potentially played by “intentionality” in the temporal binding paradigm, we introduced a cue to give a similar level of predictability to the first event (Kirsch et al. 2019, Kong et al. 2024, Saad et al. 2024), which could be an active keypress, a passive keypress, or an external sensory event (e.g. an off bulb). More precisely, in the active movement task, participants performed a voluntary key press that switched on a light bulb. In the passive movement task, the experimenter pressed the participant’s left index finger down onto the button to switch on the light bulb. In the external sensory event task, participants merely observed the appearance of a light bulb that turned on after a delay. Using Bayes factor (BF) analyses, we found no evidence of IB at any of these intervals when comparing active and passive movement tasks. Most notably, robust temporal bindings emerged when comparing both active and passive movement tasks to an externally triggered baseline task. Because these results did not differ based on whether the action outcome occurred in near or far space—a question of great interest to us—we pooled the data from both distances in our published article.

**Figure 3:**
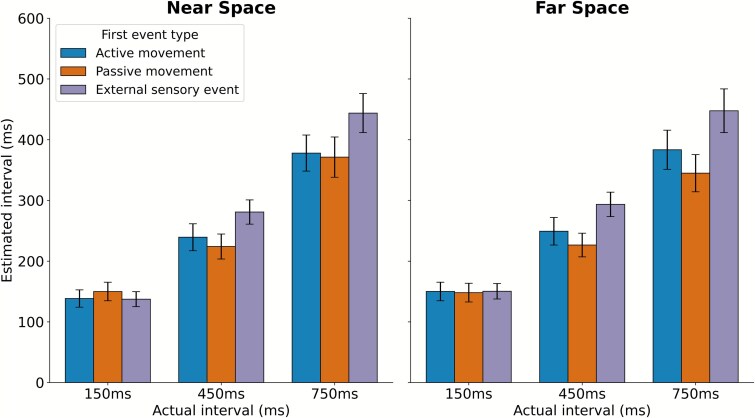
Estimated time intervals across near and far space for active movement, passive movement, and external sensory event tasks. The left panel represents results for the near-space condition (45 cm), and the right panel represents the far-space condition (4.5 m). The *x*-axis indicates the actual interval (150, 450, and 750 ms), while the *y*-axis shows the estimated interval (ms). Data are color-coded to distinguish between the first event types: blue for active trials, orange for passive trials, and purple for external sensory events. Error bars indicate the standard error of the mean (SEM).

In light of Jenkins and Obhi (2021) and Mariano et al. (2024), we reanalyzed our previous data and conducted additional BF analyses to assess whether distance could still modulate “nonintentional” binding and the perceptual shift in temporal estimation (Fig. 3). First, we report the results that concern temporal binding, analyzed separately for the near and far distances, using the same assumptions as those outlined in Kong et al. (2024). Specifically, when comparing the active and passive movement tasks, the BF indicated substantial evidence for the null hypothesis at both distances, i.e. supporting the absence of IB (Near: BF_HN(0, 122.5)_ = 0.11, RR (robustness region)_*B* < 1/3_ = [40, >3 000]; Far: BF_HN(0, 122.5)_ = 0.27, RR_*B* < 1/3_ = [100, >3000]). However, we observed a robust temporal binding effect when comparing both active and passive movement tasks to an externally triggered sensory event at both distances (Fig. 3). For the active movement versus external sensory event task, the BF indicated substantial evidence for a difference, at both distances (Near: BF_HN(0, 122.5)_ = 4.01, RR_*B* > 3_ = [9, 167]; Far: BF_HN(0, 122.5)_ = 3.29, RR_*B* > 3_ = [10, 135]). Similarly, for the passive movement versus external sensory event task, the BF showed strong evidence for a difference, at both distances (Near: BF_*U*(13.5, 122.5)_ = 24.32, RR_*B* > 3_ = [1, 882]; Far: BF_*U*(13.5, 122.5)_ = 4090, RR_*B* > 3_ = [1, >3000]).

To examine whether nonintentional temporal binding was modulated by distance, we calculated the BF by using a half-normal distribution with a standard deviation of 17.265 ms, which was the size of the distance effect on temporal interval estimation in the active task, extracted from figure 2 of Mariano et al. (2024). Using this prior, we found inconclusive evidence that spatial distance modulates the temporal binding between active movement and external event tasks (BF_HN(0, 17.265)_ = 0.43, RR_1/3 < *B* < 3_ = [1, 22]). When testing each interval separately, the evidence was also all inconclusive for all delays (150 ms: BF_HN(0, 17.265)_ = 0.50, RR_1/3 < *B* < 3_ = [1, 27]; 450 ms: BF_HN(0, 17.265)_ = 0.69, RR_1/3 < *B* < 3_ = [1, 43]; 750 ms: BF_HN(0, 17.265)_ = 0.74, RR_1/3 < *B* < 3_ = [1, 50]).

In contrast, there was substantial evidence that spatial distance modulated the temporal binding between passive movement and external event tasks (BF_HN(0, 17.265)_ = 17.74, RR_*B* > 3_ = [4, 178]), with temporal binding being smaller in near space than in far space—thus opposite to the expected distance effect. When testing each delay separately, there was also substantial evidence supporting the distance modulation in the opposite direction for the 150 ms delay (BF_HN(0, 17.265)_ = 5.896, RR_*B* > 3_ = [5, 47]). For the 450 and 750 ms delays, the evidence was inconclusive (450 ms: BF_HN(0, 17.265)_ = 1.26, RR_1/3 < *B* < 3_ = [1, 86]; 750 ms: BF_HN(0, 17.265)_ = 2.35, RR_1/3 < *B* < 3_ = [1, 398]). Because we observed a discrepancy whereby the effects reported in supplementary table S1 of Mariano et al. (2024) appear to be twice the magnitude of those shown in their figure 2, we reported an additional analysis using an alternative a priori distribution in the Supplementary Material, which does not alter our main conclusions.

However, a modulation analysis presupposes that the effect to be modulated is present. In our VR study (Kong et al. 2024), we did not observe IB in the strict sense (Active − Passive), and therefore, those data cannot adjudicate whether IB changes with distance.

In order to directly test whether spatial distance modulates temporal binding in the target literature, we computed the decisive difference-of-differences contrast ΔIB = (Active − Passive)_Near_ − (Active − Passive)_Far_ at the critical 200 ms delay for both previous studies. For Mariano et al. (2024), raw participant data were not accessible. Using their figure 2, we obtained ΔIB = −6.18 ms, and we estimated SE_Δ_ = 8.24 ms from their supplementary data. (In supplementary table S1, SE_Δ_ = 16.48 ms; as other values were twice the values reported in their figure 2, we estimated SE_Δ_ = 8.24 ms for the main BF calculation). Using a half-normal distribution with a standard deviation of 17.265 ms (the size of the distance effect on temporal interval estimation in the active task extracted from their figure 2), we found inconclusive evidence for a stronger binding effect in near space compared to far space (BF_HN(0, 17.265)_ = 0.82, RR_1/3 < *B* < 3_ = [1, 49]). An additional robustness analysis using an alternative a priori distribution is reported in the Supplementary Material, which provides the same conclusion.

For Jenkins and Obhi (2021), the raw data of 144 participants are available. In the 3D condition at 200 ms, we obtained ΔIB = −0.007022 (slider units) = −7.022 ms with SE = 0.005931 (slider units) = 5.931 ms. The BF analysis, using a half-normal distribution with a standard deviation of 17.265 ms, indicated inconclusive evidence for a stronger binding in near space compared to far space (BF_HN(0, 17.265)_ = 1.06, RR_1/3 < *B* < 3_ = [1, 62]). An additional robustness analysis using an alternative a priori distribution is reported in the Supplementary Material, which provides the same conclusion.

Together, these direct tests indicate that when the correct ΔIB contrast is applied, neither dataset supports the claim that spatial distance increases IB. Thus, we argue that the available evidence does not suggest that spatial distance modulates temporal bindings in contexts involving intentional movements - i.e. the temporal binding between active movement and passive movement or between active movement and external event baseline tasks. Indeed, no study to date has provided substantial evidence that the difference between the active task and the passive task (Mariano et al. 2024), or the external event baseline task (Jenkins and Obhi 2021), is modulated by distance. Furthermore, our study also offers inconclusive evidence for an effect of distance on “intentional binding.”

### So, what does spatial distance modulate in these studies?

Both Jenkins and Obhi (2021) and Mariano et al. (2024) reported that the shortest interval was estimated significantly shorter in near space than in far space. In Mariano et al. (2024), this effect was observed in the active movement task, while in Jenkins and Obhi (2021) it occurred in the 200 ms delay, 3D perspective condition, independently of the presence or absence of active movement.

Our primary inferential tests for agency claims are Bayesian and are conducted on the decisive ΔIB contrast in the preceding section. However, to align our summary analysis with the reporting framework used in those studies and to enable direct, like-for-like comparison of omnibus effects and simple contrasts on interval estimates (i.e. outside the intentional binding question), we also report a conventional repeated-measures ANOVA in this subsection, which here serves only to situate distance-related changes in general time perception. Specifically, we conducted a 2 × 3 × 3 repeated-measures ANOVA with the factors Distance (near, far), Task (active movement, passive movement, and external sensory event), and Delay (150, 450, and 750 ms). The results involving the Distance factor showed no significant main effect of Distance (*F*(1, 32) = 2.04, *P* = .16, partial *η*^2^ = 0.06) and no significant three-way interaction (*F*(4, 128) = 0.57, *P* = .68, partial *η*^2^ = 0.02). However, the interactions between Distance and Delay (*F*(2, 64) = 3.43, *P* = .038, partial *η*^2^ = 0.10) and between Distance and Task (*F*(2, 64) = 4.15, *P* = .02, partial *η*^2^ = 0.12) were significant. *Post hoc* analyses revealed that, independent of the task, both the 150 (*P* = .016) and 450 ms (*P* = .028) delays, but not the 750 ms (*P* = .31) delay, were perceived as shorter in near space compared to far space. Additionally, independent of delay, the intervals were perceived as shorter in near space compared to far space for the external event baseline task (*P* = .048), but not for the active (*P* = .079) and passive (*P* = .073) movement tasks.

Thus, across studies, an effect of proximity seems to emerge: the shortest delay was perceived as shorter in near space than in far space. Such shortening was only observed for the active task in Mariano et al. (2024) and it was also observed, but was independent of the task, in both Jenkins and Obhi (2021) and in our study. In addition, such shortening could also be observed for the middle delay in our work. Importantly, independent of the delay, the near versus far shortening was also observed in tasks that did not involve any movement, such as the external event baseline task in our data. Therefore, on one hand, the distance-related modulation seems to be consistently observed across studies; on the other hand, it appears to be unspecific to voluntary action execution and not limited to the shortest interval estimation, possibly suggesting that distance may broadly impact the perception of temporal durations.

These findings are also consistent with previous studies using different paradigms. For example, Anelli et al. (2015) employed a duration bisection task and found that durations were judged as shorter in near space. Petrizzo et al. (2021) extended this finding using a duration reproduction task and found systematic underreproduction of durations in near space and overreproduction in far space. In contrast, Qi et al. (2019) applied a temporal reproduction task with attentional anchoring to the hands and found that the reproduced intervals were longer when stimuli appeared near both hands. This apparent dilation in near space was attributed to prolonged disengagement of attention near the body, especially under sequential response conditions where participants had to respond with both hands; the distance effect was reduced when only one hand was used, supporting the role of attentional anchoring. Finally, spatial modulation of time perception may also impact other processes: e.g. Iachini et al. (2017) used a collision prediction task and found lower temporal discrimination accuracy and a more liberal “collision-imminent” bias in near space. Taken together, despite some divergence in the direction of duration perception (i.e. compression or dilation), these studies converge on one point: spatial distance, in and of itself, modulates the perception of time, even in the absence of voluntary action, motor signals, or causal relationships.

Although Mariano et al. measured explicit agency in their study and found no effect of distance, the task used to measure explicit agency was very simple (pressing a button to switch on a light bulb). Such simple tasks are commonly used in the field, yet they might not be sensitive enough to detect the effect of distance. Moreover, the light bulb in the “far” condition was not particularly distant, and people are accustomed to this everyday situation, as light bulbs are usually much farther from the light switch in real life. Therefore, it might be hasty to conclude that spatial distance has no effect on the explicit sense of agency. Therefore, while three studies—Jenkins and Obhi (2021), Mariano et al. (2024), and our own (Kong et al. 2024)—contribute valuable insights into the potential influence (or absence of influence) of spatial distance of an action outcome on the temporal binding and the sense of agency, further investigations are needed to establish whether spatial distance—as manipulated through depth cues, perspective, or VR—impacts the sense of agency.

## Future directions

To date, three studies—Jenkins and Obhi (2021), Mariano et al. (2024), and our own (Kong et al. 2024)—aimed to examine whether spatial distance of action outcome influences the sense of agency. Despite their methodological differences, all the studies pointed to preliminary indications that distance may influence temporal interval perception, irrespective of variations in time intervals or experimental conditions, and this effect does not appear to be linked to agency.

Currently, our lab is actively exploring new experimental paradigms to further investigate the relationship between spatial proximity, the sense of agency, and decision-making in voluntary actions (Kong et al. 2025). One key approach, already applied by Mariano et al., involves acquiring both implicit and explicit task paradigms. However, given the increasing challenges that the “intentional binding” paradigm faces as an implicit measure of agency, we would suggest using more natural, complex, goal-directed tasks (e.g. object manipulation, steering) not merely having participants press a button. This will allow for capturing both participants’ conscious and reflective sense of control over their actions and outcomes, and more implicit and automatic sensorimotor performance (Synofzik et al. 2008, Moore et al. 2012, Dewey and Knoblich 2014). This approach could involve tasks that require continuous control and adaptation (Applebaum et al. 2025), providing a more nuanced understanding of how implicit and explicit aspects of the sense of agency operate in different spatial contexts.

Advancements in VR technology offer exciting opportunities to explore how environmental contexts, whether real or simulated, interact with spatial proximity to influence agency. In VR settings, one can manipulate spatial parameters with greater precision and introduce novel scenarios, such as dynamic environments or interactions with multiple objects (Kong et al. 2017), which are not easily achievable in traditional lab settings. This will allow investigating whether and how spatially complex and immersive environments impact the sense of agency, both implicitly and explicitly. However, such designs can elicit demand-conforming responses from participants. To mitigate these effects, researchers could use a neutral, task-relevant cover story that does not disclose the study’s agency-related aim, brief pre-/post-task expectancy probes with pre-registered criteria for exclusion, and manipulation checks confirming that distance/depth cues are encoded without cueing the hypothesis. Beyond behavior, future research should also examine the neural correlates of spatially modulated agency using neuroimaging techniques, such as electroencephalography (EEG), magnetoencephalography (MEG), brain stimulation, and functional magnetic resonance imaging (fMRI). These methods will help identify the brain processes and neural correlates underlying the putative effects of spatial proximity on agency, providing a more integrated view of how spatial cognition and agency may interact.

Additionally, examining individual differences in the sense of agency would be commendable, as factors such as personality traits, cognitive styles, and motor abilities may influence how spatial proximity affects one’s perception of agency. Understanding these individual differences could have important implications for designing interventions or applications in fields such as rehabilitation, gaming, and human–computer interaction, where the sense of control over distant or virtual objects is crucial.

Finally, it would also be important to investigate whether spatial proximity influences the sense of responsibility over moral action outcomes, as the sense of responsibility is closely linked to agency (Haggard and Tsakiris 2009, Gallagher 2012). Exploring whether physical or even psychological distance affects moral responsibility over action consequences could offer insights into how spatial factors might modulate ethical decision-making and accountability in both virtual and real-world settings (Darley and Batson 1973). This line of inquiry could also build on findings in social psychology that demonstrate the impact of psychological distance on moral judgments, such as construal level theory and its effects on perceived agency and responsibility (Eyal et al. 2008, Trope and Liberman 2010).

Finally, adversarial collaborations are a promising way to resolve theoretical, empirical, and methodological disputes. A Peer Community In Registered Report (PCI RR) (https://rr.peercommunityin.org/PCIRegisteredReports/) could preregister (i) standardized stimulus, hardware and depth-cue calibration; (ii) a Bayesian analysis plan with informed priors plus equivalence testing; and (iii) blinded data processing with code-frozen pipelines. The participating labs could codevelop the Stage-1 protocol, specifying sampling rules a priori.

Together, incorporating these diverse approaches will not only deepen our understanding of whether and how spatial context modulates the sense of agency, but also advance the field by addressing methodological gaps and expanding the scope of investigation to more ecologically valid and technologically advanced settings.

## Supplementary Material

Supplementary_Material-NCONSC-2025-006_niaf066

## Data Availability

All the primary data are publicly available on the Open Science Framework repository (https://osf.io/ua3nw/).

## References

[ref1] Anelli F, Candini M, Cappelletti M et al. The remapping of time by active tool-use. PLoS One 2015;10:e0146175. 10.1371/journal.pone.0146175 26717521 PMC4696728

[ref2] Applebaum A, Netzer O, Stern Y et al. The body knows better: sensorimotor signals reveal the interplay between implicit and explicit sense of agency in the human mind. Cognition 2025;254:105992. 10.1016/j.cognition.2024.105992 39454392

[ref3] Buehner MJ . Awareness of voluntary and involuntary causal actions and their outcomes. Psychol Conscious Theory Res Pract 2015;2:237.

[ref4] Buehner MJ, Humphreys GR. Causal binding of actions to their effects. Psychol Sci 2009;20:1221–8. 10.1111/j.1467-9280.2009.02435.x 19732384

[ref5] Darley JM, Batson CD. “From Jerusalem to Jericho”: a study of situational and dispositional variables in helping behavior. J Pers Soc Psychol 1973;27:100. 10.1037/h0034449

[ref6] Dewey JA . Feelings of responsibility and temporal binding: a comparison of two measures of the sense of agency. Conscious Cogn 2024;117:103606. 10.1016/j.concog.2023.103606 37995434

[ref7] Dewey JA, Knoblich G. Do implicit and explicit measures of the sense of agency measure the same thing? PLoS One 2014;9:e110118. 10.1371/journal.pone.0110118 25330184 PMC4199671

[ref8] Eyal T, Liberman N, Trope Y. Judging near and distant virtue and vice. J Exp Soc Psychol 2008;44:1204–9. 10.1016/j.jesp.2008.03.012 19554217 PMC2701315

[ref9] Gallagher S . Multiple aspects in the sense of agency. New Ideas Psychol 2012;30:15–31. 10.1016/j.newideapsych.2010.03.003

[ref10] Gelman A, Stern H. The difference between “significant” and “not significant” is not itself statistically significant. Am Stat 2006;60:328–31. 10.1198/000313006X152649

[ref11] Gutzeit J, Weller L, Kürten J et al. Intentional binding: merely a procedural confound? J Exp Psychol Hum Percept Perform 2023;49:759. 10.1037/xhp0001110 37166936

[ref12] Haggard P . Sense of agency in the human brain. Nat Rev Neurosci 2017;18:196–207. 10.1038/nrn.2017.14 28251993

[ref13] Haggard P, Chambon V. Sense of agency. Curr Biol 2012;22:R390–2. 10.1016/j.cub.2012.02.040 22625851

[ref14] Haggard P, Tsakiris M. The experience of agency: feelings, judgments, and responsibility. Curr Dir Psychol Sci 2009;18:242–6. 10.1111/j.1467-8721.2009.01644.x

[ref15] Haggard P, Clark S, Kalogeras J. Voluntary action and conscious awareness. Nat Neurosci 2002;5:382–5. 10.1038/nn827 11896397

[ref16] Iachini T, Ruotolo F, Vinciguerra M et al. Manipulating time and space: collision prediction in peripersonal and extrapersonal space. Cognition 2017;166:107–17. 10.1016/j.cognition.2017.03.024 28554080

[ref17] Jenkins M, Obhi SS. Exploring the relationship between perceived action-outcome distance and agency: evidence from temporal binding. Conscious Cogn 2021;94:103177. 10.1016/j.concog.2021.103177 34332203

[ref18] Kirsch W, Kunde W, Herbort O. Intentional binding is unrelated to action intention. J Exp Psychol Hum Percept Perform 2019;45:378. 10.1037/xhp0000612 30570318

[ref19] Kong G, Aberkane C, Desoche C et al. No evidence in favor of the existence of “intentional” binding. J Exp Psychol Hum Percept Perform 2024;**50**:626. 10.1037/xhp0001204 38635224

[ref20] Kong G, Barlerin B, Desoche C et al. Spatial distance and temporal attentional focus modulate voluntary action preparation and awareness. bioRxiv 2025;2025.05. 20.654810. 10.1111/psyp.70280 PMC12983192 41823074

[ref21] Kong G, He K, Wei K. Sensorimotor experience in virtual reality enhances sense of agency associated with an avatar. Conscious Cogn 2017;52:115–24. 10.1016/j.concog.2017.04.018 28501698

[ref22] Kühn S, Brass M, Haggard P. Feeling in control: neural correlates of experience of agency. Cortex 2013;49:1935–42. 10.1016/j.cortex.2012.09.002 23067726

[ref23] Mariano M, Stanco G, Graps DI et al. The sense of agency in near and far space. Conscious Cogn 2024;120:103672. 10.1016/j.concog.2024.103672 38452630

[ref24] Moore JW, Obhi SS. Intentional binding and the sense of agency: a review. Conscious Cogn 2012;21:546–61. 10.1016/j.concog.2011.12.002 22240158

[ref25] Moore JW, Middleton D, Haggard P et al. Exploring implicit and explicit aspects of sense of agency. Conscious Cogn 2012;21:1748–53. 10.1016/j.concog.2012.10.005 23143153 PMC3566545

[ref26] Pálfi B, Dienes Z. Why Bayesian “evidence for H 1” in one condition and Bayesian “evidence for H 0” in another condition does not mean good-enough Bayesian evidence for a difference between the conditions. Adv Methods Pract Psychol Sci 2020;3:300–8. 10.1177/2515245920913019

[ref27] Petrizzo I, Castaldi E, Anobile G et al. Time and numerosity estimation in peripersonal and extrapersonal space. Acta Psychol 2021;215:103296. 10.1016/j.actpsy.2021.103296 33765520

[ref28] Qi Y, Wang X, He X et al. Prolonged subjective duration near the hands: effects of hand proximity on temporal reproduction. Psychon Bull Rev 2019;26:1303–9. 10.3758/s13423-019-01614-9 31144134

[ref29] Saad L, McCurry JM, Trafton G. Action and outcome predictability impact sense of agency. In: *Proceedings of the Annual Meeting of the Cognitive Science Society*, 2024; 46. Austin, TX, USA.

[ref30] Suzuki K, Lush P, Seth AK et al. Intentional binding without intentional action. Psychol Sci 2019;30:842–53. 10.1177/0956797619842191 31023161

[ref31] Synofzik M, Vosgerau G, Newen A. I move, therefore I am: a new theoretical framework to investigate agency and ownership. Conscious Cogn 2008;17:411–24. 10.1016/j.concog.2008.03.008 18411059

[ref32] Trope Y, Liberman N. Construal-level theory of psychological distance. Psychol Rev 2010;117:440. 10.1037/a0018963 20438233 PMC3152826

[ref33] Zapparoli L, Seghezzi S, Devoto F et al. Altered sense of agency in Gilles de la Tourette syndrome: behavioural, clinical and functional magnetic resonance imaging findings. *Brain Communications* 2020a;2:fcaa204. 33409491 10.1093/braincomms/fcaa204 PMC7772095

[ref34] Zapparoli L, Seghezzi S, Zirone E et al. How the effects of actions become our own. Sci Adv 2020b;6:eaay8301. 32937445 10.1126/sciadv.aay8301 PMC7458439

