# Supplementary material for: The sense of agency in near and far space: where do we stand?
Source: Neurosci Conscious. 2026 Jan 27;2026(1):niaf066. doi: 10.1093/nc/niaf066 (PMC12840585; doi:10.1093/nc/niaf066)
Supplement: Supplementary_Material-NCONSC-2025-006_niaf066 [file supplementary_material-nconsc-2025-006_niaf066.docx]

**Supplementary Material**

for *The sense of agency in near and far space: where do we stand?*

by Gaiqing Kong, Marine Vernet, and Alessandro Farnè

**Bayes Factor analysis with an a priori based on the supplementary table S1 of Mariano et al. (2024)**

Reanalysis of the distance effect on temporal binding in the data of Kong et al. (2024)

Based on values from supplementary table S1 of Mariano et al. (2024), the size of the distance effect on temporal interval estimation in the active task was 34.5 ms - twice the size reported in their figure 2. Re-calculating the BF by using a half-normal distribution with a standard deviation of 34.5 ms, we found substantial evidence that spatial distance did not modulate the temporal binding between active movement and external event tasks (BF_HN(0,34.5)_ = 0.23, RR_B<1/3_ = [23, >3000]). When testing each interval separately, there was also substantial evidence supporting the absence of distance modulation for the 150 ms interval (BF_HN(0, 34.5)_ = 0.28, RR_B<1/3_ = [28, >3000]). For the 450ms and 750 ms delays, the evidence was inconclusive (450ms: BF_HN(0, 34.5)_ = 0.41, RR_1/3<B<3_ = [1, 43]; 750ms: BF_HN(0, 34.5)_ = 0.47, RR_1/3<B<3_ = [1, 50]). In contrast, there was substantial evidence that spatial distance modulated the temporal binding between passive movement and external event tasks (BF_HN(0,34.5)_ = 13.36, RR_B>3_ = [4, 178]), with temporal binding being smaller in near space than in far space – thus opposite to the expected distance effect. When testing each delay separately, there was also substantial evidence supporting the distance modulation in the opposite direction for the 150 ms delay (BF_HN(0, 34.5)_ = 3.93, RR_B>3_ = [5, 47]). For the 450 ms and 750 ms delays, the evidence was inconclusive (450ms: BF_HN(0, 34.5)_ = 0.77, RR_1/3<B<3_ = [1, 86]; 750ms: BF_HN(0, 34.5)_ = 2.46, RR_1/3<B<3_ = [1, 398]).

Reanalysis of the distance effect on temporal binding in the data of Mariano et al. (2024)

Using their supplementary table S1, we obtained ΔIB = -12.36 ms (i.e., twice the value reported in their figure 2) with SE_Δ_ = 16.48 ms. Using a half-normal distribution with a standard deviation of 34.5 ms (the size of the distance effect on temporal interval estimation in the active task extracted from their supplementary material, i.e., twice the value reported in their figure 2), the results was virtually identical: BF_HN(0, 34.5)_ = 0.82, RR_1/3<B<3_ = [1, 99].

Reanalysis of the distance effect on temporal binding in the data of Jenkins & Obhi (2021)

A BF analysis, using a half-normal distribution with a standard deviation of 34.5 ms (the value indicated in the supplementary material of Mariano et al. (2024)) also provided inconclusive evidence for a stronger binding in near than in far space (BF_HN(0, 34.5)_ = 0.59, RR_1/3<B<3_ = [1 62]).
